# An Update on the Ketogenic Diet, 2012

**DOI:** 10.5041/RMMJ.10072

**Published:** 2012-01-31

**Authors:** Ayelet Halevy, Lilach Peleg-Weiss, Roni Cohen, Avinoam Shuper

**Affiliations:** The Institute of Pediatric and Adolescent Neurology, Schneider Children’s Medical Center of Israel, Petach-Tikva, Israel and the Sackler School of Medicine, Tel Aviv University, Tel Aviv, Israel

**Keywords:** Epilepsy, ketogenic diet

## Abstract

The ketogenic diet has been in use for the last 90 years, and its role in the treatment of epilepsy in the pediatric population has been gaining recognition. It can be helpful in many types of epilepsies, even the more severe ones, and has a beneficial effect on the child’s alertness and cognition, which can be impaired by both the condition and the medications needed for controlling it. Parental compliance is good in spite of the inconveniences inherent in following the diet. The significant advancements in understanding the nature of the diet are in better defining when its use is contraindicated and in validating its application in severe epilepsies in infancy, such as infantile spasms. Although most neurologists do not consider it as being the preferred first-line therapy, it is often a reasonable option when two medications have already failed.

## INTRODUCTION

Ninety years after its introduction as an anti-epileptic treatment,^1^,^2^ and after periods of ups and downs in its use, the ketogenic diet (KD) has found its proper place in clinical practice. The idea of manipulating diets for therapeutic purposes has been around for centuries. For instance, fasting was used in the treatment of epilepsy since Biblical times (Matthew 17:5–21). The first modern reports of its use in the medical literature were by Guelpha in 1911 and Conklin in 1921.^3^ KD has been used to treat epilepsy in children since 1921 with little variation until recent years.^4^ The original protocol using a high-fat, low-carbohydrate diet was created at the Mayo Clinic in Rochester, MN, USA, and popularized eight decades later by the Johns Hopkins Hospital in Baltimore, MD, USA.^5^

The KD is comprised mostly of fats, with low protein and low-as-possible glucose levels, combined with caloric and fluid intake restriction. In terms of weight, 1 gram of glucose and protein is added for every 3 and 4 grams of fat, respectively. The diet is intended to replace glucose as the main energy source in the brain with ketone bodies, a product of fatty acid degradation. Studies have shown that the KD has the potential to decrease significantly the severity and number of seizures in epileptic children.^1^ However, the diet is difficult to maintain, and children often feel hungry, frustrated, and depressed. Any intake of cake or candy can lead to seizures. Thus, although parents generally prefer the diet over anti-epileptic drugs (AEDs), which have potential side effects, even the most enthusiastic ones may have trouble adhering to it, leading to a high attrition rate.^1^,^6^

The diet has been largely promoted by the Pediatric Neurology team of Johns Hopkins Hospital, headed by Dr JM Freeman, together with Drs EPG Vining and E Kossoff and others.^1^,^7^,^8^ A systematic review of 26 published papers written on the use of KD in epileptic children concluded that there is evidence to support the cautious use of KDs in children with refractory epilepsy.^3^

We use the classic Johns Hopkins protocol at the Schneider Children’s Medical Center of Israel, a tertiary university-affiliated medical facility. This review discusses the indications and contraindications for the use of the KD, its effect on seizure number and severity, electroencephalographic (EEG) tracings, cognition and alertness levels, and its application in young infants with severe forms of epilepsy.

The KD has been used worldwide despite the occasional difficulties associated with it.^9^ There are some issues specific to Israel, as mentioned in Kossoff and McGrogan’s paper.^9^ The Israeli medical centers in Tel Hashomer and Holon had enrolled about 50 patients, and the authors described the issues uniquely relevant to their populations. Many families, especially Orthodox Jewish ones, are reluctant to use medications and are willing to try alternative measures if possible. They also need to contend with the caveat of consuming meat with milk products in order to observe the laws of *kashrut*. Thus, fish (with gills) and egg recipes can include heavy whipping cream, but those with meat must not. Bread used for religious purposes (e.g. challah as part of the Sabbath meal ritual and exclusive matzah consumption during Passover) is not suitable for a 4:1 ratio diet, while fruits, vegetables, and olive oil, which are plentiful and popular in Israel, are encouraged. Finally, if the father is a descendant from the priestly lineage (a “cohen”) and is therefore forbidden to enter a place that may hold dead bodies, the KD may have to be started on an outpatient basis, without a supervised fast.^9^

## INDICATIONS AND CONTRAINDICATIONS

The inherent difficulties in maintaining the KD have narrowed its use to older children with severe, intractable epilepsy who have already failed at least two trials with AEDs (presumably a measure of intractability^10^) or infants with severe epileptic disorders, which are often associated with some degree of psychomotor retardation, who are fed via a gastric tube. The KD may also be beneficial for adults with epilepsy, but apparently to a lower extent.^1^ Contraindications to the use of the diet have been well established.^11^ The free fatty acids (FFAs) that result from the diet are transported into the mitochondria across the membrane by carnitine, facilitated by carnitine palmitoyltransferase (CPT I and II) and carnitine translocase. The load of FFA in cases of deficiency of these factors cannot be handled and can lead to severe deterioration, whereupon the diet is contraindicated.^11^ In a similar way, beta-oxidation defects within the mitochondria are contraindications to fasting or following the KD. Porphyria is also considered a contraindication.^10^ Even though these disorders are relatively rare, patients must be screened for them before initiating the KD.

In children, 20%–40% of those treated with the KD reportedly showed a >90% reduction in seizure frequency, and a further 20%–60% showed a >50% improvement.^6^ This wide range of response reflects the wide range of types of epilepsy, the variability of potential seizure control in a given patient, and other factors associated with use of the diet, such as family cooperation.^6^ Predicting the patients who will or will not respond to a KD is even more difficult than predicting for the effectiveness of AEDs, for which the relationship to seizure type is better defined. Even patients with localization-related epilepsy may sometimes improve with the diet.^12^ Therefore, in the absence of a clear contraindication,^11^ the KD may be applied for any child or infant in whom its use is reasonable,^6^ regardless of seizure type. It is also important to note that there is no clear contraindication to use the KD together with AEDs or vagal nerve stimulation, and, indeed, the KD will generally be added to a regimen that includes AEDs.^1^,^11^

### Teaching case I

A 15-month-old infant was brought to our service for the treatment of a mixed seizure disorder consisting of myoclonic seizures, infantile spasms (IS), and generalized tonic-clonic seizures. He also showed severe psychomotor retardation. The seizures had failed to respond to multiple AEDs, and at the time of referral the patient was already being treated with valproic acid. The seizures were very frequent, warranting urgent treatment. Vigabatrin was started but led to significant side effects. Therefore, we decided to try the KD.

Urinalysis showed high concentrations of acyl glycines (hexanoylglycine and suberylglycine) and increased levels of dicarboxylic acids, without significant ketonuria. One study of acylcarnitine derivatives showed a mild increase in C10 and C8 carnitine, compatible with medium-chain acyl-CoA dehydrogenase (MCAD) deficiency,^13^ an absolute contraindication to the use of KD. However, the clinical picture was not typical for this abnormality. A second possibility was that these abnormalities were secondary to valproate-induced inhibition of fatty acid oxidation. The valproic acid was discontinued, and all parameters normalized after 1 week. At that point, we felt that it was safe to initiate the KD, which led to some decrease in seizure frequency for several months, making it possible for us to at least taper the vigabatrin dose.

The obvious lessons learned from this child are: always rule out the rare contraindications before initiating the diet, even when the clinical presentation does not support the presence of a contraindication. Biochemical changes induced by intake of valproic acid can mimic those of a mitochondrial disorder,^13^ thus, awareness of potential effects of it as well as of other AEDs that are already in use is critical. In this case, although the metabolic abnormalities were valproic-acid-related, they did not allow for the use of the KD before they had been excluded by withdrawal of the medication.

## SPECIFIC CONDITIONS TREATABLE WITH THE KD

The KD has been found to be the most appropriate treatment for glucose transporter 1 deficiency and pyruvate dehydrogenase deficiency.^1^,^11^ Other epileptic conditions, including tuberous sclerosis complex, Rett syndrome, severe myoclonic epilepsy of infancy (Dravet syndrome), and specific mitochondrial disorders, also respond to the diet.^14^ One study noted a 40%–50% seizure-free response rate in patients with myoclonic-astatic epilepsy (Doose syndrome), which is higher than values reported for AEDs.^15^ Another report suggested that the KD may be more effective than AEDs for Lennox–Gastaut syndrome, and the authors recommended that it be considered for early use in affected patients.^16^ In terms of seizure type, success appears to be lower in patients with complex partial seizures^1^,^12^ or epileptiform discharges in the temporal region.^12^ Neal et al.^17^ reported that there was no significant difference in the efficacy of the treatment between symptomatic generalized or symptomatic focal syndromes. In their study, the mean percentage of baseline seizures was significantly lower in the diet group than in the controls after 3 months (*P* < 0.0001). Specifically, 38% of the subjects in the diet group had a >50% seizure reduction compared with 6% of the controls (*P* < 0.0001), and 7% in the diet group had a >90% seizure reduction compared with 0% of the controls (*P* = 0.0582).^17^ The conclusion of Keene’s review was that, overall, the estimated rate for obtaining complete seizure control was 15.6% and that one-third of the studies reported a >50% reduction in the number of seizures.^3^

## EFFECT OF THE KD ON COGNITION

In addition to its effect on the number and severity of seizures as well as on EEG activity, the KD has the potential to make the child cognitively better and more alert.^1^,^18^ This is particularly important in epileptic disorders that mostly affect cognition and alertness, such as electrical status epilepticus during slow-wave sleep (ESES). Indeed, the risk of psychomotor retardation in certain epileptic disorders, such as modified hypsarrhythmia, is a major consideration in favor of using the KD. Prolonged status epilepticus, both convulsive and non-convulsive, is also of special interest. Non-convulsive status epilepticus poses a unique problem because it is not associated with overt seizures but rather with a significant decline in linguistic ability, leading to a severe, often irreversible, deterioration in scholastic performance.^19^–^21^

### Teaching case II

**Figure 1:**
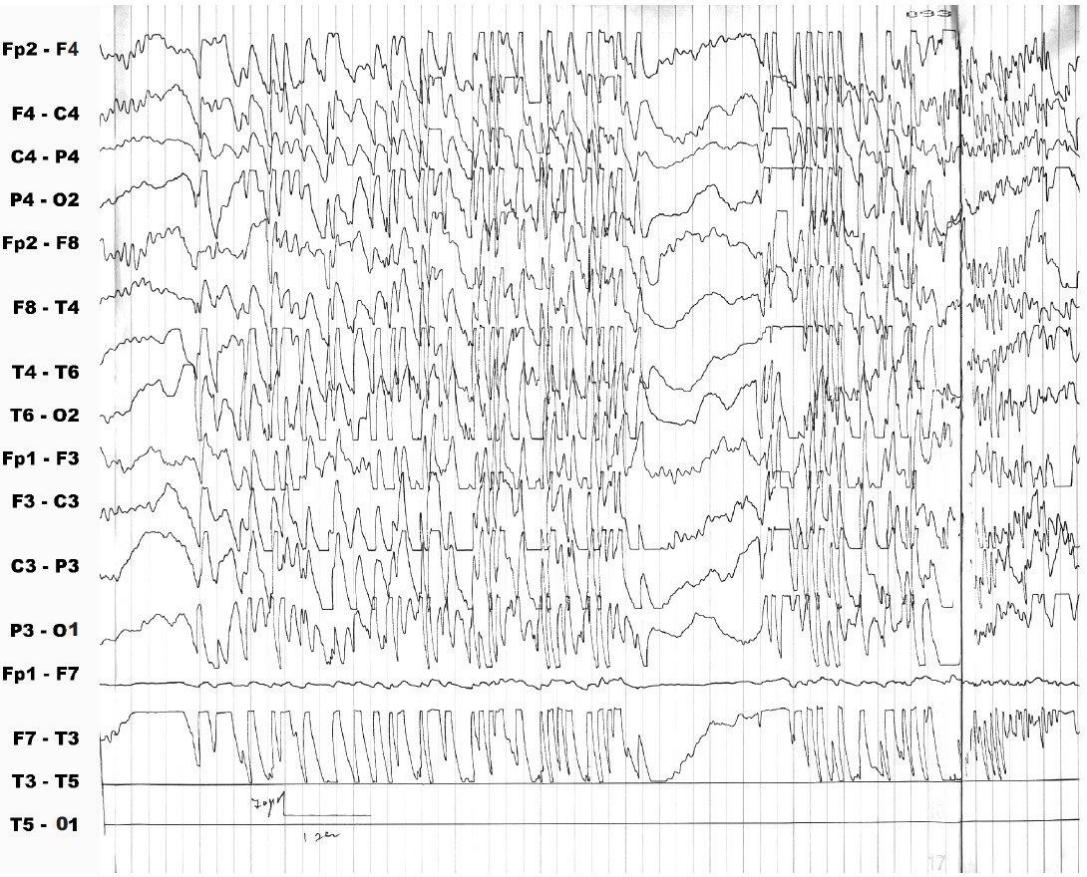
**Electroencephalographic recording showing non-convulsive status epilepticus.**

**Figure 2:**
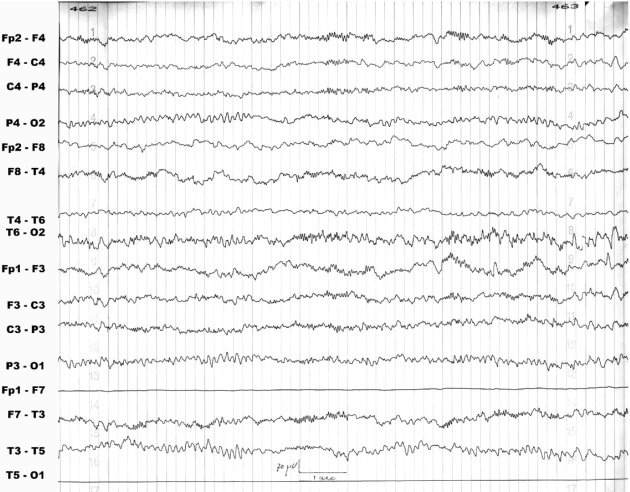
**Marked improvement of the electroencephalographic recording (EEG) 1 month after initiation of the diet in a patient on maintenance with prednisone, which had failed to normalize the EEG.**

An 8-year-old girl was referred to our center with a history of seizures of about 2.5 years’ duration. The initial seizures were generalized tonic-clonic type, and occurred only during sleep. The EEG study at the time was suggestive of idiopathic photosensitive occipital epilepsy of childhood. Treatment consisted of valproic acid with the later addition of sulthiame. This led to an improvement in seizure severity but not frequency. A learning disability and an attention deficit disorder were first manifested during the course of treatment. By age 7 years, the EEG tracing was compatible with ESES. The administration of clobazam led to the disappearance of both the seizures and the ESES pattern. However, shortly thereafter, while the patient was fully seizure-free, the EEG pattern switched to non-convulsive status epilepticus (Figure 1). The child’s alertness decreased, her scholastic problems continued, and she started exhibiting behavioral difficulties. Levetiracetam was of no benefit, and administration of intravenous immunoglobulin exacerbated the EEG findings. Intravenous pulse methylprednisolone successfully normalized the EEG but not the patient’s cognitive/behavioral state, and the non-convulsive status epilepticus recurred during a trial to taper the steroids. The patient was started on the KD. Prompt improvement was noted in the EEG findings (Figure 2) as well as in cognition, alertness, and behavior. Interestingly, ketosis was maintained during steroid treatment. With time, it was necessary to increase the dose of steroids, but the parents felt that given its clear beneficial effects, the diet should be continued despite some resistance from the child.

## USE OF THE KD IN INFANTILE EPILEPSY

The KD was found applicable for use in infantile epilepsies.^22^ The most prominent types of infantile epilepsies are IS, ranging from West syndrome to modified hypsarrhythmia. IS are characterized by myoclonic seizures that manifest clinically by clusters of sudden flexor or extensor jerks. The EEG pattern of hypsarrhythmia may be associated with psychomotor developmental arrest. The prognosis, especially for modified hypsarrhythmia, is usually very unfavorable.

Treatment for infantile seizures traditionally consists of adrenocorticotropic hormone (ACTH) and prednisone. However, these medications have variable side effects, and they can be used only for a limited period should spasms recur. Vigabatrin has received much attention for its efficacy in IS, but it can lead to some visual dysfunction. Other AEDs, such as topiramate, lamotrigine, zonisamide, valproate, clonazepam, and ganaxolone, have limited efficacy in this setting.

The KD offers an alternative therapeutic approach. The experience thus far, as summarized in recent studies by Kossoff et al.,^23^–^25^ is encouraging. Those authors reported that of 104 children with infantile spasm treated with the KD, 64% showed a >50% improvement after 6 months, including those who stopped the diet before then. One-third of the infants had prolonged periods free of spasms, and some became spasm-free permanently.^25^ Accordingly, treatment with a liquid KD in 12 infants as part of one study and 61 infants in another^26^ yielded a >90% reduction in the number of seizures. In 2010, The Infantile Spasm Working Group released guidelines for use of the KD:

“The ketogenic diet may be an option in drug-resistant epilepsy as an adjunct to pharmacologic therapy. The diet may work by enhancing γ-aminobutyric acid synthesis and improves energy utilization in the brain. Currently, there is insufficient class I evidence to recommend the ketogenic diet as a first-line intervention [for infantile spasm].”^27^

During the last 2 years, our Pediatric Neurology Service has treated more than 35 epileptic infants with the KD in the form of liquid formula, either orally or via a nasogastric tube. About one-half of them were dropped from the treatment because of unresponsiveness or side effects, such as dyslipidemia, feeding difficulties, and weight loss. About 70% of the remaining patients have shown a >90% reduction in the number of seizures, while 23% had no improvement. Infants with severe epilepsy syndromes other than IS may also be expected to benefit from the KD, including those with Ohtahara syndrome, early-onset myoclonic epilepsies, migrating partial epilepsy of infancy, and Dravet syndrome.^22^

The most daunting challenge is the use of the diet in cases of infantile myoclonic encephalopathies, from infantile spasms through severe myoclonic encephalopathy of infancy to myoclonic-astatic epilepsy and others. The influence of the diet lies within the spectrum of marked improvement in seizure control and cognitive state, to failure of the diet and inability of the parents to withstand its difficulties. The diet may be beneficial in all types of epilepsy, but the rate of success is totally unpredictable. For example, the diet was followed for 2 years and found to be helpful in a 5-year-old child with intractable complex partial seizure, a condition for which it is not expected to be beneficial. In contrast, it failed in two infants with tuberous sclerosis (length of treatment 3 and 6 months), a condition for which it was supposed to be helpful. This unpredictability of outcome motivates us to consider that a trial of the KD diet is worth the effort whatever the underlying diagnosis.

## PROGNOSIS OF INFANTS ON THE KD

The KD is usually well tolerated by the infants who are receiving oral formula, but it may be difficult to maintain in older children. The reasons for patients having discontinued the diet were often not clearly stated in the papers reviewed by Keene, but, when they were, the most frequently cited were the lack of efficacy of the diet and of compliance, not because of side effects.^3^ The most frequent side effects reported by Neal et al. at the 3-month review were constipation, vomiting, lack of energy, and hunger.^17^ In contrast, the review of 26 articles by Keene concluded that adverse events were not frequent and that vomiting (5.5%) and elevated serum lipid levels (2.6%) were the most common ones.^3^ Other rare side effects are acidosis, renal stones, gallstones, hypoglycemia, dehydration, elevated liver enzymes, protein loss enteropathy, and death. For the group of these papers, the length of time the patients had remained on the diet was 80% for at least 3 months, 60.6% for 6 months, and 35% for a year or more.^3^

The reported prognosis after treatment cessation varied. It is unclear what the ideal weaning timing and speed of the KD should be, and the resultant risk of seizure worsening has not been established. In a retrospective review by Worden et al.^8^ of 183 children who discontinued the KD at Johns Hopkins Hospital, the speed of discontinuation was categorized into immediate (<1 week), quick (1–6 weeks), or slow (>6 weeks) rates. Those authors found no significant difference in the incidence of seizure worsening between the three discontinuation rates. The conclusion drawn by the authors was that there is no increased risk of seizure exacerbation with rapid KD discontinuation. Children who had improved by 50%–99% and were receiving more anticonvulsants were at the highest overall risk. Discontinuing the KD over weeks rather than months appears safe.^8^

Patel et al. looked at the long-term outcome of 101 children with refractory epilepsy treated by KD with a median time since discontinuing the KD of 6 years (range 0.8–14 years).^28^ Few children (8%) still preferred to eat high-fat foods. 52% responder rate (>50% seizure reduction) was reported at KD discontinuation, and 79% were similarly improved (*P* = 0.0001) at the time of the research completion. While 96% of the parents or children reported that they would recommend the KD to others, only 54% would have started it before trying anticonvulsants.^28^ Less favorable results were found by Hemingway et al.:^29^ of the 150 patients in their cohort, 13% were seizure-free, an additional 14% had a 90%–99% decrease in the number of their seizures, 29 were free of medications, and 28 were on only 1 medication, and 15 remained on the diet.

The prognosis of specific epilepsies treated with KD depends on the type. For example, the outcome at 3 months after initiation of the KD in patients with intractable childhood epilepsy as a result of focal malformation of cortical development was that 61.7% showed a >50% reduction in seizure frequency, including 44.7% who became seizure-free. Of the 21 patients with complete seizure control at 3 months, 76.2% had successfully completed the diet for 2 years without relapse, and 47.6% remained seizure-free after cessation of the diet (mean follow-up 3 years and 10 months), including 1 patient who remained seizure-free with additional medication after a relapse.^30^ A second example is Dravet syndrome: of the 24 patients who were placed on the KD and followed-up for a minimum of 2 years, 66.6% remained on the diet and 12.5% became seizure free, 62.5% had a 75%–99% decrease in the number of seizures, and the remaining 25% had a 50%–74% decrease in the number of seizures.^31^ Considering the severity and refractivity of seizures in patients with Dravet syndrome, the fact that 12 of 16 children who remained on the diet had a significant reduction in the number of seizures indicates that the KD is currently an interesting therapeutic option.^31^ A third example is IS. Hong et al.^25^ reported 104 infants among whom a spasm improvement of >50% was seen in 64% at 6 months and 77% after 1–2 years, while 37% became spasm-free for at least 6 months (a median of 2.4 months since starting the KD). In addition, 62% showed improvement in development, 35% had improvement on their EEGs, and 29% were able to reduce the number of concurrent anticonvulsants. Adverse effects were noted in 33%, of which 6% had diminished linear growth. Older age at onset of IS and fewer prior anticonvulsants were considered as being more likely to be associated with a >90% spasm improvement at 6 months. The authors concluded that the KD is an efficacious therapy for IS in approximately two-thirds of treated patients and that it should be strongly considered after failure of corticosteroids and vigabatrin.^25^

## MODIFICATION OF THE KD

A growing body of evidence demonstrated that dietary therapies for epilepsy, including new modifications (classic KD, medium-chain triglyceride diet, modified Atkins diet, and low-glycemic-index treatment) are highly effective, with approximately 30%–60% of children having at least a 50% reduction in the number of seizures after 6 months of treatment.^32^

During a fasting state, the body passes through various phases of hormonal and metabolic adaptation in an attempt to spare protein breakdown and to draw on the energy reserves of body fat. The muscles and other tissues progressively switch their energy source from glucose to free fatty acids. β-Oxidation of these fatty acids results in the formation of acetyl-CoA, which is then converted into ketone bodies (acetoacetate and b-hydroxybutyrate) in the liver mitochondria. Ketone bodies, in contrast to fatty acids, are able to pass across the blood–brain barrier, and, as their levels rise in the blood, they are increasingly utilized for energy by the brain, heart, and muscle. It was suggested that a diet high in fat and low in carbohydrates might mimic this beneficial effect of fasting. A restriction of dietary carbohydrate would limit glucose supply, and, as fat is metabolized to ketone bodies, these would be used as the alternative fuel. The exact differences between these diets are detailed in Neal and Cross’s review.^33^ In one trial that included children randomized to both classical and medium-chain triglyceride protocols, there was no difference in efficacy between these two types of KDs at 3, 6, or 12 months.^34^

*Medium-chain triglycerides (MCT):* MCT are more ketogenic than long-chain-triglycerides because they generate more ketones per unit of energy when metabolized. The MCT diet has a lower proportion of fat and a greater proportion of protein and carbohydrate35^35^ thus allowing more food choices.^36^,^37^ The classical and modified MCT KDs are equally effective, and differences in tolerability are not statistically significant.^11^

*Modified Atkins diet (MAD):* The KD team at Johns Hopkins Hospital modified the Atkins diet by removing the aim of achieving weight loss, extending the induction phase indefinitely, and specifically encouraging fat consumption. Compared with the classic KD, the MAD places no limit on calories or protein, and the lower overall ketogenic ratio (approximately 1:1) does not need to be consistently maintained by all meals of the day. The MAD does not begin with a fast, or with a stay in hospital, and requires less dietitian support than the KD. Carbohydrates are initially limited to 10 g per day in children and 20 g per day in adults, and their numbers are increased to 20–30 g per day after a month or so, depending on the effect on seizure control or tolerance of the restrictions. Like the KD, the MAD requires vitamin and mineral supplements, and children are carefully and periodically monitored at outpatient clinics.^24^ The MAD reduced seizure frequency by >50% in 43% of patients who tried it and by >90% in 27% of those patients.^36^ Few adverse effects have been reported, although cholesterol is increased and the diet has not been studied long-term.^24^ In spite of being based on a smaller data set (126 adults and children from 11 studies over 5 centers), these results from 2009 compare favorably with the traditional KD.^36^

*Low glycemic index treatment* (LGIT) is an attempt to achieve the stable blood glucose levels seen in children on the classic KD while using a much less restrictive regime. The hypothesis is that stable blood glucose may be one of the mechanisms of action involved in the KD,^8^ which occurs because the absorption of the limited carbohydrates is slowed by the high fat content.^38^ Although it is also a high-fat diet (with approximately 60% calories from fat),^38^ the LGIT permits the consumption of more carbohydrates than either the classic KD or the MAD, i.e. ∼40–60 g/day.^36^ However, the types of permissible carbohydrates are restricted to those that have a glycemic index <50. Like the MAD, the LGIT is initiated and maintained at outpatient clinics and does not require precise weighing of food or intensive dietitian support. Both are offered at most centers that run KD programs and are often the primary dietary therapy for adolescents in some centers.^11^ Short-term results for the LGIT indicate that approximately one-half of the patients experience a >50% reduction in seizure frequency at 1 month, with overall figures approaching that of the KD. The data from one center’s experience with 76 children (up to the year 2009) also indicate fewer side effects than the KD and indicate that it is better tolerated, with more palatable meals.^36^,^39^

## CONCLUSION

The KD may be considered a potentially potent treatment for epilepsy in the pediatric population. Although the factors for predicting which patients will respond are still unknown, even children and infants with the more severe types of seizures may benefit. Contrary to the views of Kossoff et al.,^35^ we believe the KD is a complicated therapeutic modality and therefore inappropriate as the first-line choice. We suggest that clinicians first try medication and evaluate the patient’s response. They should then consider adding the KD to improve cognition and alertness, and to synergize the anti-epileptic effect of the drug. The pros of the diet would very likely outweigh the cons if at least two types of medication fail and the epilepsy is considered intractable.

## Abbreviations:

ACTH: adrenocorticotropic hormone;
AEDs: anti-epileptic drugs;
FFA: free fatty acids;
EEG: electroencephalogram;
ESES: electrical status epilepticus during slow-wave sleep;
KD: ketogenic diet;
LGIT: low glycemic index treatment;
MAD: modified Atkins diet;
MCT: medium-chain triglycerides.
